# Multimodal Imaging Evaluations of Focal Choroidal Excavations in Eyes with Central Serous Chorioretinopathy

**DOI:** 10.1155/2016/7073083

**Published:** 2016-06-29

**Authors:** Yao Wang, Zhi-Qing Chen, Wei Wang, Xiao-Yun Fang

**Affiliations:** Eye Center, Second Affiliated Hospital, Medical College of Zhejiang University, Zhejiang Provincial Key Lab of Ophthalmology, Number 88 Jiefang Road, Hangzhou 310009, China

## Abstract

*Purpose*. To investigate the prevalence and characteristics of focal choroidal excavation (FCE) concurrent with central serous chorioretinopathy (CSC) using multimodal imaging.* Methods*. This was a retrospective single-institution study. Clinical features and multimodal imaging findings were analyzed in eyes with CSC and FCEs, using imaging methods including optical coherence tomography (OCT), OCT angiography (OCTA), fluorescein angiography (FA), indocyanine green angiography (ICGA), fundus autofluorescence (FAF), and multispectral imaging.* Results*. Seventeen patients (4.8%) with 21 FCEs (19 eyes) were found among 351 consecutive Chinese patients with CSC. Chronic CSC represented 47.1% of those cases. Window defects in 12 lesions identified through FA and hypoautofluorescence in 13 lesions identified through FAF revealed retinal pigment epithelial attenuation. Choroidal hemodynamic disturbances characterized by localized filling defects at the excavation and circumferential hyperperfusion were validated by both ICGA and OCTA, which were similar to the angiographic features of normal chronic CSC. The hyperreflective tissue beneath FCE, observed on B-scan OCT, presented as intensive choroidal flow signals on OCTA.* Conclusions*. FCE is not uncommon in patients with CSC. Multimodal imaging suggested that the aberrant choroidal circulation might be a contribution factor for leakage from the dysfunctional retinal pigment epithelium at the area of excavation.

## 1. Introduction

Focal choroidal excavation (FCE) was first described by Jampol et al. in 2006, as an unusual choroidal excavation in the fovea with an intact overlying retina, detected by time-domain optical coherence tomography (OCT) [1]. Subsequent application of spectral-domain OCT (SD-OCT) has elucidated the clinical and more detailed morphological characteristics of FCE. These studies indicate that FCE is a choroidal abnormality without scleral involvement (absent of posterior staphyloma or scleral ectasia), which appears in two patterns: nonconforming and conforming [2–11]. Recent studies using swept-source OCT used deep wave imaging to better visualize the choroid and sclera in the area of FCE [12–14]. However, the etiology and clinical implications of FCE remain debatable. Most people consider FCE a congenital choroidal malformation, but a few acquired cases have been reported [3, 9, 15–18]. Initially, FCE was considered a relatively stationary choroidal lesion and an incidental finding in patients with either no symptoms or a slight visual disturbance. Later studies have increasingly suggested a close association between FCE and multiple vision-threatening choroidal vascular diseases, including central serous chorioretinopathy (CSC), choroidal neovascularization (CNV), or idiopathic polypoidal choroidal vasculopathy (PCV) [2–11, 13, 14].

Several studies reported that the incidence of CSC in patients with FCE ranged from 4.8% to 24.4%, far higher than incidence of CSC in normal people [3, 7–10, 12, 19, 20]. And the incidence of FCE in patients with CSC was reported to range from 2.8% to 7.8% [4, 5, 14]. Choroidal vascular disturbances in eyes with FCE were observed in indocyanine green angiography (ICGA) images, such as focal choroidal filling defect, venous dilation, and choroidal hyperpermeability, which were also typical findings in CSC [10, 19, 21]. Despite a plausible close relationship, the role of FCE in the occurrence of CSC is still unconfirmed. Suzuki et al. postulated that choroidal circulatory disruption and atrophic RPE at the FCE lesion might be related to CSC complicated by FCE [5].

Advances in OCT technique always further our knowledge about FCE. Optical coherence tomography angiography (OCTA) is a new, noninvasive, dyeless, depth-resolved technique, which enables visualization of the choriocapillaris and retinal microvasculature by combining vascular flow visualization using motion contrast and structural information [22, 23]. Hence, we supposed that OCTA could provide additional information about circulation in eyes with FCE concurrent with CSC. The aim of this study was to evaluate the prevalence, clinical, and multimodal imaging features of FCE concurrent with CSC in Chinese patients, which may therefore contribute to better understanding the underlying pathophysiology of the disease. The possible correlation between FCE and the pathogenesis of CSC has been studied with particular interest.

## 2. Materials and Methods

The Ethics Committee at the Second Affiliated Hospital of the Medical School of Zhejiang University approved this study, which was conducted in accordance with the tenets of the Declaration of Helsinki. We performed a retrospective review of 351 consecutive Chinese patients (mainland of China) with CSC who visited the eye center at the Second Affiliated Hospital of the Medical School of Zhejiang University between January 2014 and December 2015. Patients with other macular abnormalities such as CNV and angioid streaks and history of eye trauma, infection, inflammation, tumor, scleral staphyloma, or vitrectomy were excluded from the study. All study participants underwent comprehensive ocular examinations, including autorefractometry, best-corrected visual acuity (BCVA) measurement with a Snellen chart, slit-lamp biomicroscopy, and intraocular pressure measurement. Fundus examinations included indirect ophthalmoscopy, fundus photography (FP) (TRC-NW8F; Topcon Corp., Tokyo, Japan), fluorescein angiography (FA), ICGA, and fundus autofluorescence (FAF) imaging (HRA Spectralis; Heidelberg Engineering, Heidelberg, Germany). The SD-OCT images were obtained using Cirrus HD-OCT with enhanced depth imaging (EDI) OCT (Carl Zeiss Meditec, Dublin, CA, USA) or RTVue XR Avanti AngioVue OCT (Optovue, Inc., Fremont, CA, USA). Several patients underwent OCTA (OptoVue RTVue XR Avanti AngioVue; Optovue, Inc., Fremont, CA, USA) and multispectral imaging (MSI) examination (RHA; Annidis, Ottawa, Canada).

FCE was defined as a macular excavated area in the choroid, along the retinal pigment epithelium (RPE)/Bruch membrane complex line on OCT scans [14]. FCEs were classified as the “nonconforming type” if there was separation between the photoreceptor tips and the RPE and the “conforming type” if there was no separation [3, 13]. If a nonconforming FCE converted to the conforming type after the resolution of subretinal fluid (SRF), it was defined as conforming type for statistical analyses. FCEs were also classified as foveal or extrafoveal depending on whether the foveal center was located within the excavated area [3]. Three-dimensional (3D) en face imaging at the RPE level was constructed to observe the stereo shape of FCE and avoid the possibility of missing multiple lesions.

The choroidal thickness and greatest width and depth of FCE were manually measured with the built-in caliper tool on SD-OCT. Subfoveal choroidal thickness (SFChT) was defined as the distance from the outer border of the RPE to the inner scleral surface. When FCE was located within the fovea center, SFChT was measured from the uninvolved adjacent RPE layer. Choroidal thickness under the excavation was measured from the outer border of the lowest tip of the excavated RPE layer to the inner scleral surface [3, 14].

CSC was diagnosed on the basis of medical history, serous retinal detachment (SRD) detected through the fundus examination and OCT, and characteristic appearance detected through FA. Eyes showing only 1 or few specific angiographic leakage points at the RPE level were classified as classic CSC. Eyes with broad areas of granular hyperfluorescence detected through FA associated with indistinct areas of leakage were classified as chronic CSC [3].

We used a newly developed SD-OCT device (OptoVue RTVue XR Avanti AngioVue; Optovue, Inc., Fremont, CA, USA) to obtain split-spectrum amplitude decorrelation angiography (SSADA) images. SSADA is a clinically feasible, commercialized technique that can visualize capillary blood flow, but without determination of the flow direction [22]. The angiographic features at the FCE lesion and surrounding area were analyzed and compared with FA and ICGA images.

Multispectral imaging (MSI) is an improved technique using different light wavelengths from light-emitting diodes, ranging from 550 (green) to 850 nm (infrared), to examine the layers of the retina and choroid progressively [24]. MSI was performed in 4 patients to give a preliminary comparison with other fundus examinations.

The measured visual acuity was converted to a logarithm of the minimum angle of resolution (LogMAR) for statistical analysis. Statistical analysis was performed using the Statistical Package for the Social Sciences (SPSS 16.0; SPSS Inc., Chicago, IL, USA). Wilcoxon Signed Ranks test and Mann-Whitney *U* test were used to compare continuous variables. Fisher's exact test was used to compare the categorical variables. Differences were considered statistically significant when *P* values were less than 0.05.

## 3. Results

### 3.1. Demographics and Clinical Features

Seventeen patients (4.8%) with FCEs were found in 351 consecutive Chinese patients with CSC, including 11 males and 6 females (ratio of 1.83 : 1). Twenty-one FCEs were identified in 19 eyes (11 right/8 left). Two patients (11.8%) had bilateral FCE lesions, and 2 patients (11.8%) had 2 distinct lesions in the same eye. Seventeen eyes with 18 FCEs were concurrent with CSC, while the other 2 eyes with 3 FCEs were asymptomatic. Patients' demographic and clinical features are summarized in Table 1. The mean patient age was 44.3 ± 10.6 years (range, 21–65 years). The best-corrected visual acuities (BCVA) ranged from 20/100 to 20/20. The mean refractive error (spherical equivalent) was −1.18 ± 2.30 diopters (D) (range, from 2.25 to −4.75 D), and 11 eyes (61.1%) were myopic. Age-related macular degeneration with CNV was found in 1 patient's fellow eye and CSC in 2 different patients' fellow eyes. Four patients were recurrent cases. Systemic conditions were generally unremarkable.

### 3.2. Fundus Photograph, Autofluorescence, and Multispectral Imaging Features

**Figure 1:**
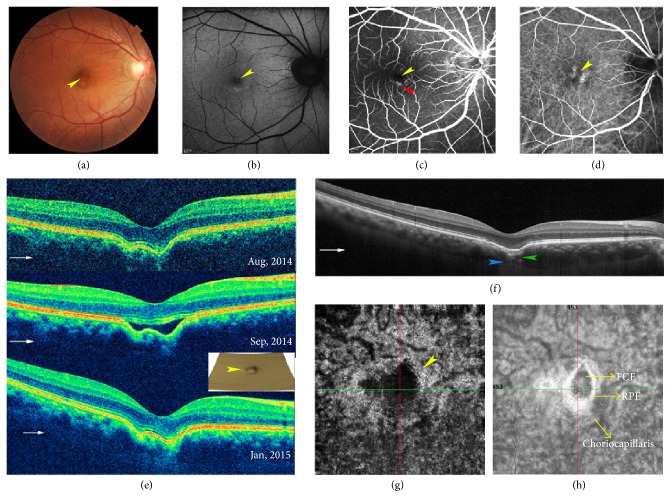
Images from a 38-year-old man with FCE in the right eye combined with classic CSC (patient 1). The patient presented with metamorphopsia which lasted for just one day. (a) Color FP shows a yellowish FCE lesion in the fovea area. (b) AF shows a small hyperfluorescent FCE lesion. (c) Early-phase FA shows the hypofluorescent FCE lesion (yellow arrowhead) and the adjacent hyperfluorescent leakage point (red arrowhead). (d) Mid-phase ICGA shows the slight hypofluorescent FCE lesion surrounded by two hyperfluorescent areas of choroidal hyperpermeability. (e) FCE converts from conforming to nonconforming, and finally back to conforming in SD-OCT images, along with the progression of CSC. The small reconstructed 3D en face OCT image by segmentation of RPE shows the umbilicate excavation with a small bulge in the center, which appears as two conterminous excavations in B-scan OCT. (f) The EDI-OCT image shows thinning ellipsoid zone, hyperreflective tissue and thinning choroid beneath FCE (green arrowhead), suprachoroidal space (blue arrowhead), and slight inward chorioscleral interface without any ectasia. The morphology of FCE differs from image (e), because the scanning line deviates from the center of FCE. (g, h) FCE presents as a dark flow signal-absent area, surrounded by a hyperperfused area in the default choriocapillaris slab of OCTA (g), and as a hyporeflective area surrounded by the hyperreflective RPE in the corresponding en face structural OCT image (h). The intersection of green line and red line represents the macular center. The yellow arrowheads point to FCE lesion in all images. The thin white arrows indicate OCT scan direction.

**Figure 2:**
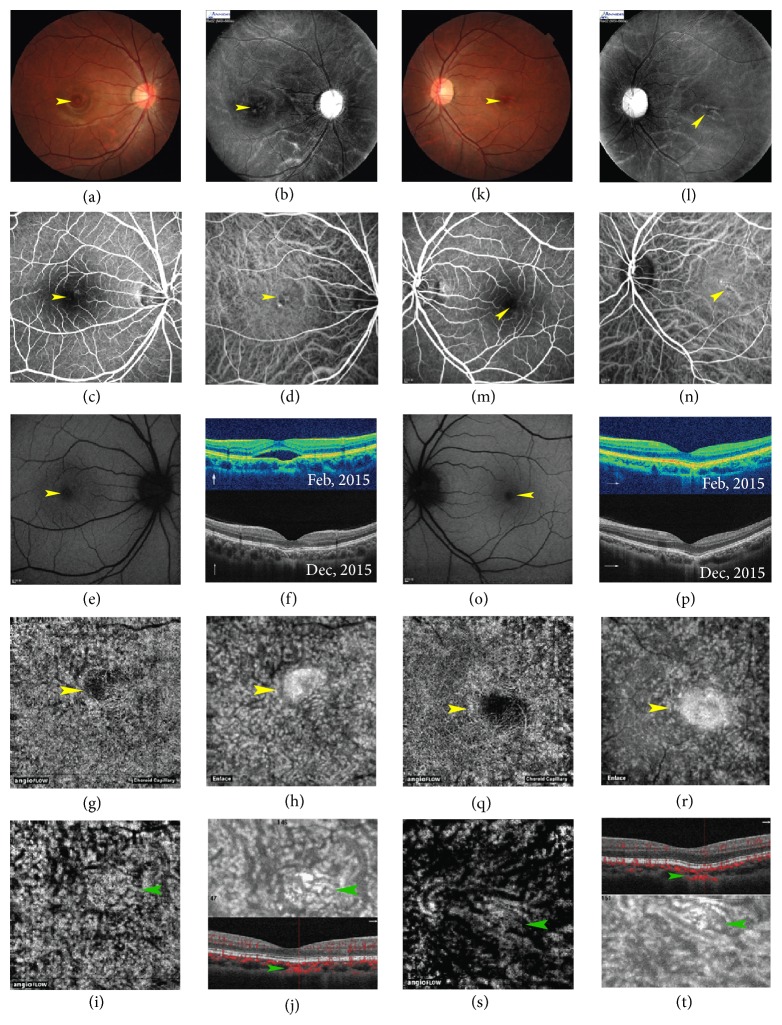
Images from a 21-year-old woman with bilateral FCEs combined with chronic CSC in the right eye (patient 7). The patient presented with micropsia which lasted for one week. (a~j) right eye. (k~t) Left eye. (a, k) Color FPs show round yellowish FCE lesion (right)/reddish FCE lesion (left) in the fovea area. (b, l) MSI 660 nm images show well-defined dark fusiform FCE lesion (b, right)/grayish FCE lesion with blurred margin surrounded by dilated choroidal vessels (l, left). (c, m) Mid-phase FA images show slight window defect in FCE lesions in both eyes. (d, n) Mid-phase ICGA images show hypofluorescent FCE lesions in both eyes (surrounded by dilated choroidal vessels in the left eye), similar to MSI images. (e, o) FAF images show small hypoautofluorescence lesions in both eyes. (f, p) FCE converts from nonconforming to conforming with the resolution of retinal detachment in SD-OCT images in the right eye (f). The morphology of both FCEs does not differ much in the 10-month follow-up duration. Hyperreflective tissues can be seen beneath the FCEs with the obviously thinning choroid in both eyes. (g, h, q, r) FCEs present as the flow signal-absent area surrounded by hyperperfused areas in the default choriocapillaris slab of OCTA (g, q). Images (h, r) show FCEs morphology in the corresponding en face structural OCT. (i, j, s, t) Hyperreflective tissue beneath FCE in B-scan OCT presents as intensive flow signals in the choroid segment of OCTA (i, s) and as a hyperreflective plaque in the corresponding en face structural OCT (j upper, t lower). Dilated choroidal vessels surrounding the FCE can be seen in the left eye (s, t lower). (i, j upper) 63 *μ*m to 91 *μ*m below the RPE reference in the right eye; (s, t lower) 100 *μ*m to 129 *μ*m below the RPE reference in the left eye. (j lower, t upper) B-scan OCT angiogram shows the intensive choroidal flow (in red) beneath the FCE, which is at the same site as the hyperreflective choroidal tissue. The green arrowheads point to the hyperreflective choroidal tissue beneath FCE. The yellow arrowheads point to FCE lesions in all images. The thin white arrows indicate OCT scan direction.

**Figure 3:**
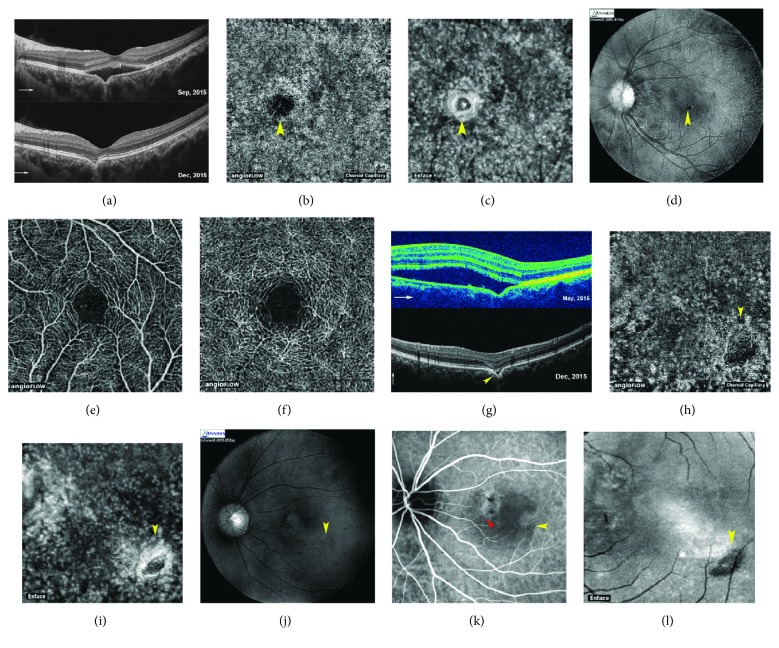
Images from a 39-year-old woman with FCE combined with chronic CSC in the left eye (patient 16) and a 43-year-old man with FCE combined with classic CSC in the left eye (patient 13). Nonconforming FCEs in both patients remained nonconforming despite the resolution of SRD. BCVA of both patients were 20/20. (a~f) Patient 16. (g ~ l) Patient 13. (a, g) The two cone-shaped FCEs remain nonconforming in B-scan OCT, and a hyperreflective material (a) and a hyporeflective space (g) can be found between photoreceptor tips and RPE, respectively, in patients 16 and 13. (b, c, h, i) FCEs present as a dark flow signal-absent area surrounded by hyperperfused area in the default choriocapillaris slab of OCTA (b, h) and as a hyporeflective area surrounded by hyperreflective RPE in the corresponding en face structural OCT images (c, i). (d, j) MSI 810 nm shows the well-defined dark teardrop FCE lesion in patient 16 (d). MSI 850 nm shows the grayish FCE lesion with blurred margin in patient 13 (j). (e, f) Superficial (e) and deep (f) retinal plexus seemed almost undisturbed in OCTA. (k) Mid-phase ICGA shows the mild hypofluorescent FCE lesion close to hyperfluorescent areas of choroidal hyperpermeability. The FCE lesion is distant from the leakage point (red arrowhead). (l) FCE can be accurately localized according to the default en face structural OCT image of outer retina slab, based on the distinct positional relationship between FCE and projection artifacts of retinal vessels. The yellow arrowheads point to FCE lesion in all images. The thin white arrows indicate OCT scan direction.

Fundus and angiographic characteristic of eyes with FCEs associated with CSC are summarized in Table 2. The presence of FCE could not be determined on clinical examination or by color FP. FCEs appeared as yellowish lesions in 10 eyes (55.6%), reddish lesions in 2 eyes (11.1%), and pigmentary mottling in 6 eyes (33.3%) (Figures 1 and 2). FAF images were available in 17 FCE lesions of 15 eyes and were classified into three groups: hypoautofluorescence (8 lesions), hyperautofluorescence (4 lesions), and a mixed area of hypo- and hyperautofluorescence (5 lesions) (Figures 1 and 2). FAF reflected the RPE status and indicated decreased RPE activity in most FCE lesions. FCEs were discernable in the MSI images ranging from 620 nm to 850 nm (available in 7 eyes). They appeared as well-defined black (3 eyes) or blurred grayish lesions (4 eyes), some of which were surrounded by dilated choroidal vessels (Figures 2 and 3). The shape of excavation in MSI images seemed different from that in the en face structural OCT images, but similar to that in ICGA images (Figure 2).

### 3.3. Spectral-Domain Optical Coherence Tomography Findings

The 3D and en face OCT scans allowed us to detect FCEs in the macular area with no omitting and to clearly visualize their morphology. Overall, 12 FCEs (57.1%) were located in the foveal area and the remaining 9 FCEs (42.9%) in the extrafoveal area. The 3D reconstructed en face images of the RPE level showed that the excavation shape varied from small and cone-like to broad irregular excavation. We noticed umbilicate excavations with small centered bulges in 3 eyes, which appeared as two conterminous excavations in the transverse B-scan OCT (Figure 1).

High-penetration OCT with a long wavelength facilitated visualization and measurement of choroidal structures. The hyperreflective choroidal tissue under the excavations reported in other studies was also found in 12 eyes, bridging between the bottom of excavation and the outer choroidal boundary, and the outer choroidal boundaries were pulled inward by the bridging tissue in 3 of those 12 eyes. The chorioscleral interface was physiologically smooth with no ectasia in all 19 eyes. Decreased choroid middle/large vessels under FCEs and dilation of large choroidal vessels adjacent to FCEs were observed in 6 eyes with hyperreflective tissues.

The greatest width and depth of FCEs were 704.4 ± 294.4 *μ*m (range, 187–1293 *μ*m) and 73.5 ± 41.3 *μ*m (range, 20–184 *μ*m). The choroidal thickness under the excavation of 18 FCE eyes (296.0 ± 106.4 *μ*m) was significantly less than the SFChT (372.4 ± 109.0 *μ*m) of the same eye (*P* = 0.000). The SFChT of 11 CSC eyes (377.00 ± 77.6 *μ*m) was thicker than that of the fellow eyes (332.5 ± 95.5 *μ*m), but there was no statistical difference (*P* = 0.075), excluding 5 patients with choroidal vascular disease or FCEs in the fellow eyes. SFChT and choroidal thickness under the excavation of 12 eyes with hyperreflective tissue beneath the excavation (338.1 ± 107.6 *μ*m and 260.8 ± 107.6 *μ*m) were both less than those of 6 eyes without hyperreflective tissue (441.0 ± 80.4 *μ*m and 372.3 ± 73.5 *μ*m), but there was no statistically significant difference in the comparison of choroidal thickness under the excavation (*P* = 0.039 for SFChT, *P* = 0.075 for choroidal thickness under the excavation).

### 3.4. Optical Coherence Tomography Angiography Findings

**Figure 4:**
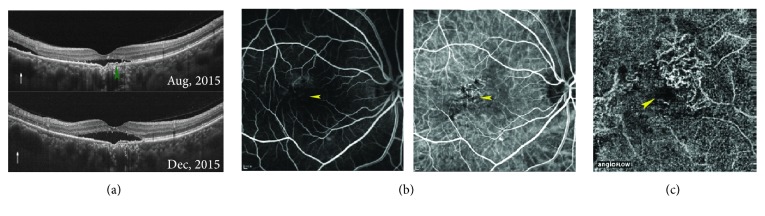
Images from a 58-year-old woman with FCE combined with chronic CSC in the right eye (patient 15). The patient presented with a two-year history of chronic CSC, and subsequent branching vascular network was observed in the affected eye. (a) SRF increases in the follow-up duration, and a double-layer sign appears with an undulated RPE profile in the vertical B-scan OCT (green arrowhead). (b) Early-phase simultaneous FA and ICGA show that a hyperfluorescent area in FA (b, right) and a discontinuous branching vascular network in ICGA (b, left) are observed superior to FCE lesion (yellow arrowhead). (c) OCTA at the level of inner choroid demonstrates a distinct and continuous choroidal neovascular network superior to FCE lesion, which is located in the same area as the vascular network in ICGA. The CNV network corresponds to the small undulations within the slight RPE detachment on B-scan OCT. Yellow arrowheads point to FCE lesion in all images. The thin white arrows indicate OCT scan direction.

OCTA images were available in 11 eyes with 13 FCEs (9 patients). The superficial and deep retinal plexus seemed almost undisturbed (Figure 3). FCE appeared as a dark flow signal-absent area, surrounded by hyperperfused areas in the default choriocapillaris slab of OCTA, and as a hyporeflective area with circumferential hyperreflective RPE in the corresponding en face structural OCT, whether the FCE was conforming or not (Figures 1, 2, and 3). In some chronic cases, it was difficult to distinguish FCE from the various focal hypoperfused lesions on the angiographic image without the assistance of B-scan OCT (Figure 4). Interestingly, the hyperreflective tissue beneath FCE in B-scan OCT presented as a plaque of intensive flow signals in OCTA and as a hyperreflective plaque in the corresponding en face structural OCT (Figure 2).

A predominant advantage of OCTA was the visualization of choroidal neovascularization. One 65-year-old woman presented with a two-year history of chronic CSC and a persistent SRD. A distinct and continuous choroidal neovascular network superior to a FCE lesion was first noticed through OCTA at the level of the inner choroid [25]. It appeared similar to a discontinuous branching vascular network in the early-phase ICGA image (Figure 4) [25].

**Figure 5:**
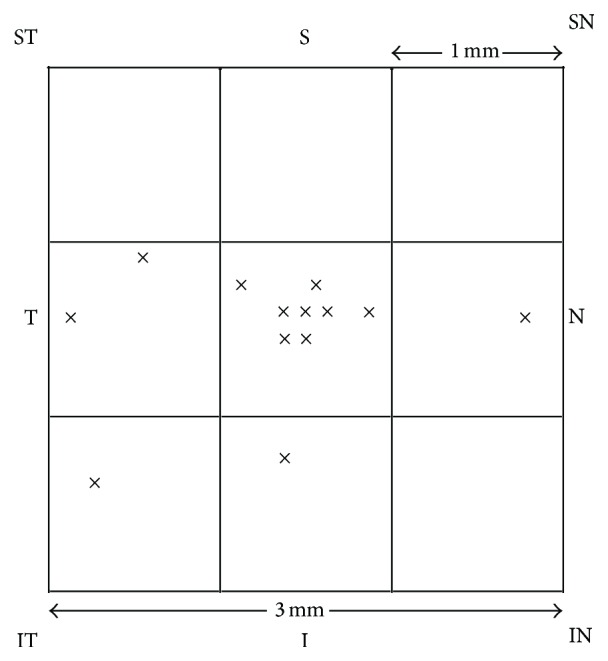
Diagram showing locations of 13 FCEs in the fovea-centered 3 mm × 3 mm square area according to the choriocapillaris slabs of OCTA in 9 patients. Each “×” represents a FCE. I = inferior; IN = inferonasal; IT = inferotemporal; N = nasal; S = superior; SN = superonasal; ST = superotemporal; T = temporal.

We noticed that FCE could be accurately localized according to the default en face structural OCT image of the outer retina slab, based on the distinct positional relationship between FCE and projection artifacts of retinal vessels. All 13 FCEs were located within the fovea-centered 3 mm × 3 mm square area, including 8 FCEs (61.5%) within the central 1 mm × 1 mm area (Figure 5).

### 3.5. Traditional Angiographic Features

All 17 patients underwent FA examinations and 10 patients underwent ICGA examinations. According to FA, 9 patients (52.9%) were diagnosed as classic CSC (leakage types: 4 ink-blot type, 4 smoke-stack type), and the other 8 patients (47.1%) were diagnosed as chronic CSC. FA showed varying degrees of hyperfluorescence and hypofluorescence related to RPE status in the FCE area. Twenty-one FCE lesions were divided angiographically into 4 groups: window defect (10 lesions, 47.6%), hypofluorescence surrounding window defect (2 lesions, 9.5%), slight hypofluorescence (5 lesions, 23.8%), and hyperfluorescence due to leakage (4 lesions, 19.1%) [9]. FCEs were located within the area of fluorescein leakage (a focal leakage point in classic CSC or a broad area of granular hyperfluorescence in chronic CSC) in 11 patients (64.7%), adjacent to the area in 4 patients (23.5%), and distant from the area in 2 patients (11.8%).

FCEs were more chiseled and discernable on ICGA images. Fourteen lesions in 12 eyes were hypofluorescent from early to late phase. Late-phase cloud-like hyperfluorescence secondary to choroidal vascular hyperpermeability was observed around FCE lesions in all eyes. Obvious choroidal vascular dilation was found in 5 eyes. In 2 patients with a lengthy history of chronic CSC, it was not easy to distinguish FCE lesions from other hypofluorescent lesions in a large area of mottled fluorescence.

### 3.6. Clinical Course

The mean follow-up duration was 12.6 ± 7.4 months. Patients' chief complaints seemed not very different from normal CSC patients, including blurred vision, micropsia, or metamorphopsia. There were 5 eyes with conforming FCEs and 14 eyes with nonconforming FCEs at the first visit. In the 5 eyes with conforming FCEs, one patient presented with metamorphopsia and was diagnosed as classic CSC by FA and subsequent emerging of SRF (patient 1). Two other eyes had a history of resolved CSC (patient 14) and a recurrent CSC with SRD distant from FCE (patient 6), respectively. The remaining two eyes were detected by routine bilateral OCT examination.

SRD including the area of FCEs existed in all 14 eyes with nonconforming FCEs. With the resolution of SRD, FCEs converted from nonconforming to conforming in 7 eyes. Three patients underwent fundus laser photocoagulation and two patients underwent half-dose photodynamic therapy (PDT). FCEs in three patients converted from nonconforming to conforming and FCEs in the other two remained nonconforming. The treatment appeared to have a limited effect on the rate of conforming FCE, compared with the rate in the patients without treatment (*P* = 1.000).

Visual acuity of most patients was restored with the resolving of SRF and the restoration of the ellipsoid zone (EZ), but one patient's vision remained 20/30 as a result of a significantly thinning EZ. At the end of follow-up, the visual acuity of 12 eyes with conforming FCEs was significantly better than that of 7 eyes with nonconforming FCEs (*P* = 0.022). During the entire follow-up period, no new FCEs formed and no obvious change of the existing FCEs was observed.

## 4. Discussion

To our knowledge, our study may be the largest case series published to date on FCE concurrent with CSC patients. In our single-institution study, the prevalence of FCEs in CSC patients was 4.8%. The rate was lower than the 7.8% reported by Ellabban et al. and the 6.0% by Luk et al., but higher than the 2.8% by Suzuki et al. [4, 5, 14]. The different rates are possibly, at least in part, due to the small sample size and also related to the OCT scanning protocol used to detect the FCE. 3D scanning protocol proved to be more sensitive in detecting FCE compared with line scanning [7]. The mean patient age was 44.3 ± 10.6 years, which was consistent with the reported age of FCE presence in patients 40 to 50 years old, and also conformed to the average age of CSC onset (45 to 51 years) [10, 20]. Although there was no sex predilection of FCE in previous studies, we found approximately twice as many males as females among our FCE/CSC patients (11 male/6 female). This ratio was surprisingly close to the previously reported male : female ratio of 2 : 1–2.5 : 1 in FCE/CSC patients [3–5, 14]. It is reasonable that the obvious male preponderance was due to the intrinsic sex predilection of CSC (male : female ratios range from 2.7 : 1 to 7 : 1) [20]. All our patients were Chinese, which was consistent with the reported Asian preponderance (90%) of FCEs [3]. As for refraction, myopia was observed in 61.1% eyes. Whether moderate myopia is a specific character of FCE is controversial; at least it is not present in some FCE/CNV patients [2, 11, 13, 14]. Therefore, the racial/ethnic, sex, and refraction predisposition still require further multicenter/race validation.

The different pathogenesis of the conforming and nonconforming types of FCE and the mechanism of their mutual conversion are still disputable. We observed two cases of conforming to nonconforming conversion due to the onset of CSC, rather than the self-progression of FCE. One presented with nonconforming FCE concurrent with CSC, whose OCT image, taken in another hospital 2 years ago, showed a conforming FCE at the same site (patient 9), as did patient 1 described in Figure 1. Interestingly, the lines of the external limiting membrane (ELM) and EZ at the detached area were always below the normal counterparts in patient 1, as if they were restricted by an uncertain force. Margolis et al. hypothesized that conforming FCE could progress to nonconforming FCE as stress on the outer retina resulted in separation of the photoreceptors tips from the apical surface of RPE [2]. The progression was much like CSC, and it was hard to make a distinction, as obvious leakage points could not be observed in many CSC cases.

Nonconforming to conforming conversion is more common, as seen in the resolution of CSC and CNV [3, 6, 7]. Lim et al. postulated that continued weakening of the choroid with age may lead to slow enlargement of the excavation and conversion from nonconforming FCE into conforming FCE [12]. It is plausible that the mutual conversion of the two FCE types is mostly dependent on the prognosis of complicated choroidal diseases, rather than the spontaneous progression of FCEs. In our study, FCEs in two eyes remained nonconforming despite the SRD regression, which had a small hyporeflective space and hyperreflective material between the photoreceptor tips and the underlying RPE, respectively (Figure 3). A possible reason for the former could be preexisting nonconforming FCE before CSC progression, or dysfunction of the pumping action resulting from RPE atrophy at the FCE lesion [3–5]. The hyperreflective material in the latter case was supposed to be unabsorbed subretinal deposits. Decreased elasticity and tension made the outer retina fail to attach to the deeply outpouching RPE layer.

It is well accepted that there is a significantly attenuating choroid beneath the excavation [14, 21]. Nevertheless, whether the uninvolved choroid is thickened or not is controversial. Margolis et al. agreed that a thick choroid might predispose patients to FCE [2, 26, 27]. Ellabban et al. thought the choroid in FCE/CSC patients is thicker than in people with normal eyes, but thinner than in CSC patients without FCE [14]. However, Lee and Obata et al. observed that the choroid thickness of FCE patients is similar to normal, but FCE patients with CSC seemed to have a thicker choroid than the FCE patients without CSC [3, 6, 10]. In our study, the SFCT of 18 FCE eyes (372.4 ± 109.0 *μ*m) was thicker than normal Chinese eyes (253.8 ± 107.4 *μ*m), which was apparently due to the CSC-related choroid thickening [20, 28]. Interestingly, bilateral FCEs were observed in patient 7 with thinner-than-average choroidal thickness (153 and 196 *μ*m) and typical choroidal hemodynamic features of FCE. We propose that the choroidal thickness may not be the key factor in the pathogenesis of FCE, but the disturbed choroidal circulation may be.

In our study, SFChT of eyes with hyperreflective tissue beneath the excavation was significantly less than that of eyes without hyperreflective tissue. It seemed that the existence of hyperreflective tissue would result in more striking RPE retraction and choroidal thinning. Ellabban et al. suspected that hyperreflective tissue represented focal scarring in the choroidal connective tissue, whereby subsequent scar contraction results in RPE retraction and FCE formation [14]. It was unexpected that hyperreflective tissue beneath the FCE presented as intensive flow signals in the choroid segment of OCTA. The nonvascular nature of the hyperreflective tissue was seemingly not supported by the OCTA findings. We speculated the hyperreflective tissue to be fibrovascular malformation in the choroid, which might be formed by abnormal vascularization in embryo period, or by subtle or subclinical choroidal inflammation [18]. These findings may be involved in the pathogenesis of FCE.

Multimodal angiographies furthered our knowledge on the circulation around the excavation. FA/ICGA imaged the flow of blood plasma or staining of vessel walls and tissue, independent of cell movement, while OCTA measured the blood flow dependent on intravascular cell movements. Therefore, these three techniques provided complementary circulation information [22]. Based on FA manifestation, chronic CSC represented 47.1% of FCE/CSC cases, which seemed remarkably higher than that reported in normal CSC patients (5%) [29]. The abnormal high proportion was also reported by Ellabban et al. (66.7%) and Lee et al. (50%) [3, 14]. In our study, FCEs in 88.2% of patients were located within or adjacent to the area of fluorescein leakage, which demonstrated the close association between FCEs and RPE dysfunctional area. Window defects identified through FA in 57.1% lesions and hypoautofluorescence identified through FAF in 76.5% lesions revealed varying degrees of RPE attenuation and dysfunction. Suzuki et al. speculated choroidal ischemic changes might cause RPE atrophy at the FCE [5].

Localized filling defects that appeared at the excavation on ICGA seemed to be related to the obviously thinning choroid tissue beneath FCE and choroidal ischemic changes [4]. Choroidal vascular hyperpermeability around the FCE lesions revealed irregular hyperperfusion status of the circumferential choroid. The aberrant choroidal circulation was validated in more detail by OCTA as a localized flow signal-absent area with circumferential hyperperfused area in the choriocapillaris slab. Hemodynamic abnormalities of FCE on OCTA were highly consistent and colocated with those on ICGA, which suggested association with the formation of FCE [4, 10]. Obata et al. suggested CSC shared highly similar characteristics with FCE in ICGA [10]. A recent study suggested foci of ischemia surrounded by reactive choroidal hyperperfusion were an important factor in the pathogenesis of normal chronic CSC, through a comparison among OCTA, ICGA, and FA [22]. The multimodal imaging findings were quite similar to our findings of FCE. Combined with the unexpected high proportion of chronic CSC in FCE/CSC patients, we supposed that the typical choroidal hemodynamic disturbances would also contribute to the leakage at the FCE site and the conversion from conforming to nonconforming FCE.

Our study was mainly limited by the small number of patients and the retrospective and cross-sectional data acquisition. We had data for only a few patients' FCE status before the onset of CSC. To further improve our knowledge of the progression of, and vascular changes in, FCE, prospective, longitudinal, and multicenter studies are required to monitor FCE during its natural course and when it is concurrent with CSC. A large-scale epidemiology study in normal asymptomatic people is needed to explicate whether the prevalence of FCE in CSC patients is equivalent to or higher than that in normal people. The fields of view on OCTA were considerably smaller than those currently used in ICGA or SD-OCT [22]; therefore, information outside the fields was missed.

## 5. Conclusions

In conclusion, FCE was a phonotype on OCT and diagnosed only by OCT. Although sex predilection of FCE was still not explicit, we found the male : female ratio seemed to be approximately 2 : 1 in FCE patients concurrent with CSC. The hyperreflective tissue beneath FCE might partly have a vascular nature based on the OCTA findings. Multimodal imaging depicted that the choroidal hemodynamic disturbances in the area of excavation were characterized by focal hypoperfusion with circumferential choroidal hyperperfusion, which were accompanied with disturbed RPE function. The similar angiographic features shared by both FCE and chronic CSC, the unusual high proportion of chronic CSC in FCE/CSC patients, and the close topographic relationship between FCE and leakage area all led us to speculate that choroidal thinning and aberrant choroidal circulation at the area of excavation might be causes for the leakage from the dysfunctional RPE, and thus for the conversion of FCE from conforming type to nonconforming type. OCTA showed significant textural changes of the choriocapillaris flow pattern in FCE/CSC cases, and further quantitative examination of choroidal blood flow would expand our knowledge on FCE.

## Figures and Tables

**Table 1:** Demographic and clinical features of eyes with FCEs associated with CSC.

Patient	Age, yrs	Sex	BCVA	Spherical equivalent, D	Excavation						SFChT, *μ*m	CSC type	Treatment
					Location	Hyperreflective tissue	FCE type	Choroidal thickness under the excavation, *μ*m	Depth, *μ*m	Width, *μ*m			
1	38	M	20/30	−0.625	Foveal	Yes	Conforming → nonconforming → conforming	183	184	1283	366	Classic	
2	40	F	20/25	−2.25	Foveal	Yes	Nonconforming → conforming	307	56	496	318	Classic	
3	42	F	NA	NA	Foveal	NA	Nonconforming → conforming	NA	134	874	NA	Chronic	
4	47	M	20/20	1.00	Foveal	Yes	Nonconforming → conforming	314	33	256	348	Classic	
5	65	M	20/20	−1.75	Foveal	Yes	Nonconforming	226	97	1293	312	Chronic	
6	42	M	20/20	−3.00	Foveal	Yes	Conforming	311	97	743	371	Classic	
7^a^ (OD)	21	F	20/20	−4.75	Foveal	Yes	Nonconforming → conforming	109	60	696	153	Chronic	
(OS)			20/20	−4.75	Foveal	Yes	Conforming	108	41	808	196		
8	60	M	20/40	2.25	Foveal	No	Nonconforming	383	47	746	409	Chronic	Laser
9	35	M	20/50	0.25	Foveal	No	Nonconforming	271	68	473	378	Chronic	
10	41	M	20/25	−2.50	Foveal	No	Nonconforming	297	44	568	349	Chronic	
11	50	M	20/20	1.50	Extrafoveal	Yes	Nonconforming → conforming	434	43	775	530	Classic	Laser
12^b^	50	M	20/20	1.00	Foveal	Yes	Nonconforming → conforming	308	100	1192	433	Chronic	Laser
					Extrafoveal	Yes	Nonconforming → conforming	302	32	496		Chronic	
13^c^	43	M	20/20	1.75	Extrafoveal	No	Nonconforming	427	119	696	537	Classic	
14	48	F	20/20	−1.75	Foveal	Yes	Conforming	129	111	913	219	Classic	
15	58	F	20/100	0.625	Foveal	Yes	Nonconforming	356	57	539	426	Chronic	PDT
16^d^	39	F	20/20	−0.50	Foveal	Yes	Nonconforming	351	76	510	385	Chronic	
17^e^ (OS)	34	M	20/20	−3.50	Foveal	No	Nonconforming → conforming	398	20	187	434	Classic	PDT
(OD)			20/20	−4.25	Foveal	No	Conforming	416	94	718	539		
					Extrafoveal	No	Conforming	458	30	530			

NA = not available; SFChT = subfoveal choroidal thickness; Laser = laser photocoagulation.

^a^Patient 7 had bilateral lesions (2 FCEs): the right eye with 1 FCE was concurrent with CSC, and the left eye with 1 FCE was asymptomatic.

^b^Patient 12 had two distinct lesions in the same eye concurrent with CSC.

^c^In patient 13, the photoreceptor tips remained separated from the RPE by a small hyporeflective space, after complete resolving of serous retinal detachment.

^d^In patient 16, the photoreceptor tips remained separated from the RPE by a small hyperreflective material, after complete resolving of serous retinal detachment.

^e^Patient 17 had bilateral lesions (3 FCEs): the right eye with 1 FCE was concurrent with CSC, and the left eye with 2 distinct FCEs was asymptomatic.

**Table 2:** Fundus and angiographic characteristic of eyes with FCEs associated with CSC.

Patient	FP	FAF	FA		ICGA		OCTA^e^		MSI^f^
			FCE	Correlation between FCE and leakage/hyper area	FCE	Surrounding choroidal hyperpermeability	FCE	Surrounding increased flow	
1	Yellowish	Hyper	Hypo	Within leakage point	Hypo	Yes (vessel dilation)	Dark	Yes	NA
2	Yellowish	Hypo	Within leakage	Within leakage point	NA	NA	NA	NA	NA
3	NA	NA	Slight WD	Adjacent to granular hyper	NA	NA	NA	NA	NA
4	Yellowish	Hypo	Hypo surrounding WD	Within leakage point	NA	NA	NA	NA	NA
5	Yellowish	NA	Slight WD	Within leakage point	NA	NA	NA	NA	NA
6	PM	Hypo	Slight WD	Adjacent to leakage point	NA	NA	NA	NA	NA
7^a^ (OD)	Yellowish	Hypo	Within granular hyper	Within granular hyper	Hypo	Yes (vessel dilation)	Dark	Yes (slight)	Well-defined black
(OS)	Reddish	Hypo	Stippled WD		Hypo	Yes (vessel dilation)	Dark	Yes	Blurred grayish
8	Yellowish	Mixed	Slight WD	Adjacent to hyper area	Hypo	Yes	NA	NA	NA
9	Yellowish	Mixed	WD	Within granular hyper	Hypo	Yes	NA	NA	NA
10	PM	NA	Within granular hyper	Within granular hyper	Hypo	Yes (vessel dilation)	NA	NA	NA
11	PM	Hypo	WD	Extra leakage point	Hypo	Yes	Dark	Yes	NA
12^b^	PM	Hypo	WD	Within granular hyper	Hypo	Yes (vessel dilation)	Dark	Yes	NA
	PM	Hypo	WD	Within granular hyper area	Hypo	Yes (vessel dilation)	Dark	Yes	NA
13	Yellowish	Mixed	WD	Extra leakage point	Hypo	Yes	Dark	Yes	Blurred grayish
14	Yellowish	NA	Slight hypo	Within leakage point	NA	NA	Dark	Yes (slight)	NA
15^c^	PM	Mixed	Hypo surrounding WD	Within granular hyper area	Hypo	Yes	Dark	Yes	Well-defined black
16	PM	Mixed	Within granular hyper	Within granular hyper area	NA	NA	Dark	Yes	Well-defined black
17^d^ (OS)	Reddish	Slight hyper	Slight hypo	Adjacent to leakage point	Hypo	Yes	Dark	Yes	Blurred grayish
(OD)	Yellowish	Slight hyper	Slight hypo		Hypo	Yes	Dark	Yes	Blurred grayish
	Yellowish	Slight hyper	Slight hypo		Hypo	Yes	Dark	Yes (slight)	Blurred grayish

NA = not available; PM = pigmentary mottling; WD = window defect; Hypo = hypofluorescence; Hyper = hyperfluorescence.

^a^Patient 7 had bilateral lesions (2 FCEs): the right eye with 1 FCE was concurrent with CSC, and the left eye with 1 FCE was asymptomatic.

^b^Patient 12 had two distinct lesions in the same eye concurrent with CSC.

^c^In patient 15, secondary choroidal neovascular network was visible on choriocapillaris slab of OCTA.

^d^Patient 17 had bilateral lesions (3 FCEs): the right eye with 1 FCE was concurrent with CSC, and the left eye with 2 distinct FCEs was asymptomatic.

^e^Dark area in OCTA means no blood flow signal.

^f^MSI: from 620 nm to 850 nm.
